# Establishment and application of a SNP molecular identification system in *Grifola frondosa*

**DOI:** 10.3389/fmicb.2024.1417014

**Published:** 2024-08-08

**Authors:** Bin Diao, Zhixiang Xu, Min Liu, Guoli Zhang, Guangyuan Wang, Yinghao Zhang, Xuemei Tian

**Affiliations:** Shandong Province Key Laboratory of Applied Mycology, College of Life Sciences, Qingdao Agricultural University, Qingdao, China

**Keywords:** KASP marker, variety identification, DNA fingerprinting, core collection, edible mushrooms

## Abstract

Germplasm resources of edible mushrooms are essential for the breeding of varieties with improved traits. Analysis of the genetic diversity of *Grifola frondosa* germplasm resources and clarification of the genetic relationships among strains can provide valuable information for the selection of breeding parents. A total of 829,488 high-quality SNP loci were screened from 2,125,382 SNPs obtained by sequencing 60 *G. frondose*. Phylogenetic analysis, PCA, and population structure analysis based on the high-quality SNPs showed that the 60 strains could be divided into five subgroups, and the clustering results were consistent with the geographical distributions of these strains. Based on high-quality SNP loci, a core collection containing 18 representative germplasm resources was constructed, and 1,473 Kompetitive Allele-Specific PCR markers were obtained. A total of 722 SNP markers in the exonic regions were screened using KASP-genotyping experiments, and 50 candidate SNP markers and 12 core SNP markers were obtained. Genetic fingerprints of *G. frondosa* germplasm resources were constructed based on the selected SNP markers; these fingerprints provide an accurate, rapid, convenient, and efficient method for the identification of *G. frondosa* germplasm resources. The results of this study have important implications for the preservation and utilization of *G. frondosa* germplasm resources and the identification of varieties.

## 1 Introduction

*Grifola frondosa* is a rare edible and medicinal mushroom that belongs to the phylum Basidiomycota and the family Polyporaceae (Dai, 2009; Dai et al., 2010). Its fruiting body is flavorful and rich in nutrients, such as proteins, minerals, polysaccharides, sterols, and triterpenes; it has also been shown to have anti-tumor, antioxidant, and immunomodulatory properties (Wu et al., 2021; Tripodi et al., 2022). The cultivation of this rare edible mushroom has increased rapidly in recent years in China because of its nutritional value, pharmacological functions, and broad market prospects.

The artificial domestication and cultivation of *G. frondosa* in China began in the 1980s. The factory production of *G. frondosa* is necessary to meet the continuous growth in demand for *G. frondosa* (Li, 2021; Li and Xu, 2022). Existing strains have mainly been domesticated in the wild or introduced from Japan; these strains have various shortcomings such as their poor adaptability, high vulnerability to being contaminated with mold, low biotransformation rate, and inability to be used for factory cultivation. These strains have been used for the cultivation of mushrooms; their poor traceability coupled with the widespread presence of mixed varieties poses a threat to the future productivity of mushroom farming operations and breeding efforts (Li et al., 2019a). Moreover, the genetic background of cultivated germplasm is narrow, and the diversity of favorable alleles in wild germplasm has not been fully utilized in breeding programs. The identification of mushroom strains is a challenge due to the low number of morphological differences between mushroom varieties. This poses challenges for the establishment of protections for edible mushroom varieties; there is thus an urgent need to develop efficient and accurate technologies for the identification of different mushroom varieties (Li et al., 2019a). There is also a need to preserve and improve existing *G. frondosa* germplasm resources; improved methods for the accurate identification of varieties are also needed, especially for the identification of specific germplasm materials and the selection of parents with desirable traits. An increasing number of technologies, including molecular marker technologies, have been used in studies of edible and medicinal mushrooms (Xu, 2006; Su et al., 2008; Liu et al., 2018; Zhang et al., 2019).

The core collection refers to a limited set of accessions that are selected to maximize the genetic diversity of the population with the lowest genetic redundancy. The core collection generally comprises a genetically representative sample of the population. Core collections have been constructed for various plants, such as *Oryza sativa* (Chung et al., 2009), *Phaseolus vulgaris* (Perseguini et al., 2015), *Lagenaria siceraria* (Wang et al., 2021b), and *Juglans regia* (Mahmoodi et al., 2021). Few core collections of fungi have been constructed; to date, core collections have been generated for the following fungal species: *Lentinus edodes* (Liu et al., 2015), *Flammulina velutipes* (Liu et al., 2018), *Pleurotus citrinopileatus Singer* (Zhang et al., 2012), and *Saccharomyces cerevisiae* (Liu et al., 2021).

Kompetitive Allele-Specific PCR (KASP) is based on touch-down PCR technology, which utilizes universal fluorescent probes for accurate double allele typing of target SNPs against a wide range of genomic DNA samples, including complex genomic DNA samples (Wang et al., 2022). KASP technology has various advantages compared with other SNP detection methods, including its high accuracy, high site adaptability, low cost, and suitability for detecting SNP sites in a large number of samples; these properties make KASP useful for the identification of crop genes, genetic diversity analysis, and fingerprinting (Li et al., 2019b; Yang et al., 2022).

The construction of DNA fingerprints based on SNP marker technology is important for ensuring their varietal specificity and utility for varieties or species authenticity issues; DNA fingerprints are also important for genetic breeding programs, the identification of germplasm resources, and the discovery of new genes (Omar et al., 2023).

Whole genome resequencing is to find the difference between sequence information by comparing with existing genomes, so that the population variation information can be quickly obtained, such as SNP, InDel, SV, CNV and other variation sites (Bau and Lu, 2017; Huang et al., 2018). Compared with other SNP detection methods, KASP technology has the advantages of high accuracy and site adaptability, relatively low cost, and suitable for high-throughput detection of a large number of samples, and has a variety of applications in genetic diversity analysis and fingerprint construction. At present, it has been widely used in the construction of fingerprint of different species, such as cabbage (Li et al., 2018), bottle gourd (Wang et al., 2021b), grape (Wang et al., 2021a) and so on.

In this study, whole-genome resequencing technology was used to screen high-quality SNP loci, establish a core collection, and develop KASP markers from *G. frondosa* germplasm resources. Principal component analysis (PCA), population genetic structure analysis, and phylogenetic analysis based on developed KASP molecular markers were used to verify their discriminatory power. Our findings will enhance the efficiency of the management and utilization of *G. frondosa* germplasm resources, provide a valuable source of genetic information, and aid the genetic breeding of *G. frondosa* in China.

## 2 Materials and methods

### 2.1 Strains, DNA extraction, and whole-genome sequencing

A total of 60 strains of *G. frondosa* were used in this study, including 49 cultivars, 3 wild strains, and 8 aerospace mutagenic strains (Table 1). The mycelia were grown in Potato Dextrose Agar (PDA) medium at 25°C until they reached maturity. Genomic DNA was extracted from mycelia using the CTAB method (Doyle and Doyle, 1987). A Nanodrop spectrophotometer and 1.0% agarose gel electrophoresis were used to assess the concentration and integrity of the DNA. After the DNA was fragmented via ultrasound. The DNA fragments were purified and end-repaired, a single “A” nucleotide was added to the 3′ ends of the blunt fragments, sequencing adapters were ligated to the A-tailed fragments, and then size selection was performed using agarose gel electrophoresis; this was followed by PCR amplification to generate sequencing libraries. The constructed libraries were first subjected to library quality control, and the qualified libraries were sequenced using the Illumina NovaSeq 6000 platform (Li et al., 2024).

**TABLE 1:** Basic information of *G. frondosa* strains.

Sample ID	Strain	Origin	Cultivar/Wild/Mutant
Gf-1	5.486	Institute of Microbiology, Chinese Academy of Sciences, Beijing, China	Cultivar
GF-2	5.404	Institute of Microbiology, Chinese Academy of Sciences, Beijing, China	Cultivar
Gf-3	ACCC50289	Sanming Mycological Institute, Sanming, Fujian, China	Cultivar
Gf-4	ACCC50477	Beijing University of Agriculture, Beijing, China	Cultivar
Gf-5	ACCC50642	Qingyuan, Zhejiang, China	Cultivar
Gf-6	ACCC50651	Institute of Plant Protection, Beijing Academy of Agriculture and Forestry Sciences, Beijing, China	Cultivar
Gf-7	ACCC50641	Institute of Edible Fungi, Shanghai Academy of Agricultural Sciences, Shanghai, China	Cultivar
Gf-8	ACCC50981	Institute of Plant Protection, Beijing Academy of Agriculture and Forestry Sciences, Beijing, China	Cultivar
Gf-9	ACCC51100	Japan	Cultivar
Gf-10	ACCC50717	Australia	Cultivar
Gf-11	GIM5.164	Sanming Mycological Institute, Sanming, Fujian, China	Cultivar
Gf-12	No.2	Beijing Ruidi Fungus Industry, Beijing, China	Cultivar
Gf-13	No.4	Beijing Ruidi Fungus Industry, Beijing, China	Cultivar
Gf-14	5.248	Shanxi Agricultural University, Taiyuan, Shanxi, China	Cultivar
Gf-15	5.626	Shanxi Agricultural University, Taiyuan, Shanxi, China	Cultivar
Gf-16	H235	Shanxi Agricultural University, Taiyuan, Shanxi, China	Cultivar
Gf-17	236	Shanxi Agricultural University, Taiyuan, Shanxi, China	Cultivar
Gf-18	238	Shanxi Agricultural University, Taiyuan, Shanxi, China	Cultivar
Gf-19	5.692	Shanxi Agricultural University, Taiyuan, Shanxi, China	Cultivar
Gf-20	240	Qingyuan Scientific Research Center of Edible Fungi, Qingyuan, Zhejiang, China	Cultivar
Gf-21	241	Liaoning Academy of Agricultural Sciences, Shenyang, Liaoning, China	Cultivar
Gf-22	HS18	Chuxiong, Yunnan, China	Cultivar
Gf-23	Hui-01	Sichuan Agricultural University, Chengdu, Sichuan, China	Cultivar
Gf-24	Hui-02	Sichuan Agricultural University, Chengdu, Sichuan, China	Cultivar
Gf-25	H247	Gutian Edible Fungi Research Institute, Fujian Agriculture and Forestry University, Fuzhou, Fujian, China	Cultivar
Gf-26	248	Hebei, China	Cultivar
Gf-27	249	Henan, China	Cultivar
Gf-28	250	Jiangsu, China	Cultivar
Gf-29	251	Laiyang, Shandong, China	Wild
Gf-30	252	Laiyang, Shandong, China	Wild
Gf-31	253	Japan	Cultivar
Gf-32	254	Fujian, China	Cultivar
Gf-33	G5	Fujian, China	Cultivar
Gf-34	256	Fujian, China	Cultivar
Gf-35	257	Sichuan, China	Cultivar
Gf-36	258	Sichuan, China	Cultivar
Gf-37	F3	Japan	Cultivar
Gf-38	261	Gaoyou, Jiangsu, China	Cultivar
Gf-39	262	Jiayu, Hubei, China	Cultivar
Gf-40	265	Laiyang, Shandong, China	Wild
Gf-41	Hui51-1	Qingdao, Shandong, China	Mutant
Gf-42	Hui51-2	Qingdao, Shandong, China	Mutant
Gf-43	Hui51-4	Qingdao, Shandong, China	Mutant
Gf-44	Hui51-5	Qingdao, Shandong, China	Mutant
Gf-45	Hui51-6	Qingdao, Shandong, China	Mutant
Gf-46	Hui51-7	Qingdao, Shandong, China	Mutant
Gf-47	Hui51-8	Qingdao, Shandong, China	Mutant
Gf-48	Hui51-9	Qingdao, Shandong, China	Mutant
Gf-49	277	Liaoning Academy of Agricultural Sciences, Shenyang, Liaoning, China	Cultivar
Gf-50	278	Liaoning Academy of Agricultural Sciences, Shenyang, Liaoning, China	Cultivar
Gf-51	279	Taian, Shandong, China	Cultivar
Gf-52	280	Taian, Shandong, China	Cultivar
Gf-53	281	Taian, Shandong, China	Cultivar
Gf-54	283	Jiayu, Hubei, China	Cultivar
Gf-55	284	Zhejiang, China	Cultivar
Gf-56	285	Qingdao, Shandong, China	Cultivar
Gf-57	286	Qingdao, Shandong, China	Cultivar
Gf-58	QX01	Shandong Academy of Agricultural Sciences, Jinan, Shandong, China	Cultivar
Gf-59	QH151	Shandong Academy of Agricultural Sciences, Jinan, Shandong, China	Cultivar
Gf-60	QH152	Shandong Academy of Agricultural Sciences, Jinan, Shandong, China	Cultivar

The raw reads obtained from sequencing were analyzed using fastqc. After removing the adapters and low-quality reads with fastp software (Chen et al., 2018), the clean reads were mapped to the reference genome using BWA-MEM software (v.0.7.17) with default parameters^1^ (Li and Durbin, 2009). Information on the sequencing depth, genome coverage, and variation among samples is shown in Table 2.

**TABLE 2:** Data for whole genome-sequenced strains.

Sample ID	Read Number	BaseNumber	GC (%)	Q30 (%)	MappedProper Reads	Mean Genome Coverage	AverCoverDepth
Gf-1	9874838	1481225700	45.56	92.24	8448904 (85.56%)	91.79%	37.19
Gf-2	11171390	1675708500	45.64	92.16	9589480 (85.84%)	91.85%	42.17
Gf-3	8092238	1213835700	46.44	92.09	6953276 (85.93%)	91.67%	30.67
Gf-4	8671194	1300679100	44.92	91.91	7252170 (83.64%)	91.59%	32.22
Gf-5	8294046	1244106900	44.31	91.64	7024494 (84.69%)	91.70%	30.94
Gf-6	10868916	1630337400	45.69	92.24	9330194 (85.84%)	91.93%	40.99
Gf-7	9420008	1413001200	45.34	92.33	8071118 (85.68%)	91.77%	35.49
Gf-8	8924340	1338651000	44.42	92.22	7526766 (84.34%)	91.91%	33.28
Gf-9	10826484	1623972600	45.93	89.9	9267620 (85.60%)	91.99%	40.73
Gf-10	11079256	1661888400	44.15	92.87	9288650 (83.84%)	91.92%	41.13
Gf-11	9633182	1444977300	44.43	91.7	8173926 (84.85%)	91.77%	36.01
Gf-12	8215584	1232337600	45.79	92.52	7063556 (85.98%)	91.60%	31.12
Gf-13	8290964	1243644600	46.23	91.76	7144224 (86.17%)	91.65%	31.43
Gf-14	9928706	1489305900	45.44	91.33	8505872 (85.67%)	91.85%	37.4
Gf-15	10268808	1540321200	44.55	92.3	8660004 (84.33%)	91.82%	38.29
Gf-16	8884854	1332728100	44.48	92.6	7546278 (84.93%)	91.59%	33.24
Gf-17	8069370	1210405500	46.05	92.29	6961938 (86.28%)	91.34%	30.88
Gf-18	9226136	1383920400	44.81	91.29	7822930 (84.79%)	91.78%	34.53
Gf-19	10270426	1540563900	44.52	91.9	8747092 (85.17%)	91.56%	38.77
Gf-20	11123200	1668480000	45.31	92.59	9545118 (85.81%)	91.61%	42.25
Gf-21	8540026	1281003900	45.38	89.82	7309588 (85.59%)	91.83%	32.08
Gf-22	7511332	1126699800	46.24	92.41	6505892 (86.61%)	91.54%	28.56
Gf-23	6923008	1038451200	44.76	93.18	5939690 (85.80%)	90.28%	26.66
Gf-24	8375764	1256364600	44.37	92.37	7151142 (85.38%)	90.54%	32.09
Gf-25	9655174	1448276100	47.31	92.26	8412000 (87.12%)	91.85%	36.89
Gf-26	10280486	1542072900	46.85	91.48	8888632 (86.46%)	91.95%	39.03
Gf-27	10234356	1535153400	46.06	92.47	8814222 (86.12%)	91.82%	38.74
Gf-28	9772212	1465831800	46.25	92.4	8412332 (86.08%)	91.80%	37.01
Gf-29	10329944	1549491600	47.94	91.66	8922992 (86.38%)	94.06%	38.49
Gf-30	9560820	1434123000	46.27	92.4	8372358 (87.57%)	91.80%	36.84
Gf-31	8907098	1336064700	46.84	91.12	7704216 (86.50%)	91.79%	33.88
Gf-32	10372996	1555949400	47.05	91.2	9001320 (86.78%)	91.90%	39.52
Gf-33	8748032	1312204800	46.83	90.1	7572702 (86.56%)	91.82%	33.26
Gf-34	11331124	1699668600	44.78	89.86	9630594 (84.99%)	91.01%	43.14
Gf-35	10312590	1546888500	45.95	89.4	8848310 (85.80%)	91.94%	38.88
Gf-36	10610344	1591551600	46.81	88.98	9173218 (86.46%)	92.00%	40.24
Gf-37	11446970	1717045500	46.62	90.51	9921430 (86.67%)	91.99%	43.36
Gf-38	11050756	1657613400	45.98	89.67	9488066 (85.86%)	91.96%	41.59
Gf-39	10590756	1588613400	46.84	90.28	9191102 (86.78%)	91.97%	40.25
Gf-40	10117846	1517676900	47.74	89.93	8900684 (87.97%)	92.87%	38.61
Gf-41	9872204	1480830600	46.53	90.61	8542536 (86.53%)	91.89%	37.44
Gf-42	9679276	1451891400	47.01	90.12	8449262 (87.29%)	91.84%	36.93
Gf-43	11073554	1661033100	46.04	89.05	9540958 (86.16%)	91.97%	41.78
Gf-44	11250656	1687598400	46.02	88.9	9678754 (86.03%)	91.98%	42.4
Gf-45	7902618	1185392700	44.35	88.61	5672944 (71.79%)	91.35%	25.01
Gf-46	10220188	1533028200	45.9	89.68	8807686 (86.18%)	91.90%	38.59
Gf-47	8637108	1295566200	46.52	89.43	7496116 (86.79%)	91.70%	32.81
Gf-48	10186440	1527966000	46.68	90.7	8847892 (86.86%)	91.91%	38.69
Gf-49	9044604	1356690600	45.8	90.9	7829438 (86.56%)	91.72%	34.28
Gf-50	9183504	1377525600	46.99	90.13	8012700 (87.25%)	91.88%	35
Gf-51	8826824	1324023600	44.27	89.56	7452412 (84.43%)	92.42%	32.68
Gf-52	9149606	1372440900	43.82	89.93	7768126 (84.90%)	91.23%	34.47
Gf-53	8596442	1289466300	44.86	89.8	7367838 (85.71%)	92.66%	32.1
Gf-54	10917974	1637696100	46.68	89.75	9458114 (86.63%)	91.98%	41.38
Gf-55	9026964	1354044600	46.04	89.92	7784676 (86.24%)	92.23%	33.98
Gf-56	9090704	1363605600	45.63	89.98	7845780 (86.31%)	91.32%	34.74
Gf-57	8807818	1321172700	45.54	90.4	7614748 (86.45%)	91.33%	33.66
Gf-58	8958728	1343809200	46.11	91.45	7763704 (86.66%)	91.37%	34.18
Gf-59	9083952	1362592800	46.04	90.28	7818086 (86.06%)	91.72%	34.22
Gf-60	10040764	1506114600	45.24	89.97	8565814 (85.31%)	91.78%	37.51

### 2.2 SNP detection and annotation

Localization analysis of Clean Reads in the reference genome was performed using the GATK (version: v4.1.4.1) software toolkit, and the Mark Duplicate tool in Picard software was used to remove duplicate reads derived from PCR (McKenna et al., 2010). GATK HaplotypeCaller was used to generate raw variants (SNPs and InDels). The raw SNPs were filtered using GATK VariantFiltration with the following parameters: “QD < 2.0, MQ < 40.0, FS > 60.0, MQRankSum < −12.5, and ReadPosRankSum < −8.0”. A DNA raw variation database containing 2.12 million SNPs was generated. SnpEff was used to obtain annotation information on the variant loci from the reference genome (Cingolani et al., 2012). High-quality SNPs were screened from the entire database using bcftools and vcftools software based on the following criteria: Average coverage depth > 5 ×, Minor allele frequency (MAF) > 0.05, AverageQ > 30, Minimum integrity > 0.9 (Danecek et al., 2011).

### 2.3 Population genetic analysis and core collection development

Phylogenetic trees were constructed based on high-quality SNPs using the neighbor-joining (NJ) method, and the trees were visualized using Fig Tree v1.3.1 software (Oliveira et al., 2020). PCA was performed using GCTA (v1.940) software (Yang et al., 2011). The genetic structure of populations was analyzed using Admixture (v1.3.0) software based on the number of subgroups or ancestors and the similarity among populations; a CV error diagram was made to determine the optimal K-value (Alexander et al., 2009). Core Hunter software was used to screen the core collection, and the representativeness of the core collection was assessed by calculating the Shannon-Weaver diversity index, Nei’s gene diversity index, and PIC (Thachuk et al., 2009). Principal component analysis and cluster analysis were conducted to analyze the constructed core collection (Wang et al., 2021b).

### 2.4 KASP marker design and genotyping

KASP markers were screened from high-quality SNPs using the following criteria (Yang et al., 2022): (1) SNP sites were conserved in sequences greater than 50 bp before and after the DNA strand on the chromosome; (2) average coverage depth > 5 ×, minor allele frequency (MAF) > 0.05, AverageQ > 30, minimum integrity > 0.9, and the SNP is a double-allele variant; (3) the sequences 100 bp upstream and downstream of the SNP marker were mapped to the reference genome using BLAST software (version: 2.10.1+), and markers at multiple locations in the alignment were removed; and (4) markers had a polymorphic information content (PIC) > 0.2. All PIC calculations were performed in Excel using the following formula:


$$PIC\;=\;1-\sum_{i\;=\;0}^{l}P_{i}^{2}-\sum_{i\;=\;1}^{l-1}\sum_{j\;=\;i+1}^{l}2p_{i}^{2}p_{j}^{2}$$

where P_*i*_ and P_*j*_ are the frequency of occurrence of the two SNP alleles in all tested accessions, and l is the number of samples (Botstein et al., 1980; Zhang et al., 2020).

The primer design software Primer 3 was used to design the SNP markers, which were converted into KASP markers. When designing the KASP primers, other mutation sites were avoided, and only SNP sites for which primers were successfully designed were considered qualified KASP markers (Zhang et al., 2020). Primers were designed using the following parameters: (1) GC content < 60%; (2) Melting temperature (T_*m*_) between 55 and 61°C; and (3) PCR product size no larger than 120 bp. Primers with FAM- or VIC-tails were synthesized by Sangon Biotech (Shanghai) Co., Ltd. A universal fluorescent tag was added to the 5′ end of the primers F1(FAM): GAAGGTGACCAAGTTCATGCT and F2(VIC): GAAGGTCGGAG. The newly synthesized primers were diluted to 10 μM with 1 × TE buffer (pH 8.0) and then mixed with upstream primer 1: upstream primer 2: downstream universal primer at a ratio of 1:1:3; this mixture was then added to the machine, with 1.25 μL of primer mixture added to every 5 μL of the reaction system. PCR was performed in a total volume of 5 μL containing 1.25 μL of template DNA (50 ng⋅μL^–1^), 2.5 μL of 2 × KASP Master Mix, and 1.25 μL of primer mix. The membrane of the 96-well PCR reaction plate was sealed, shaken, and centrifuged to ensure that the reaction system was homogeneously mixed; after centrifugation, the PCR reaction was performed using the CFX ConnectTM Real-Time System, Bio-Rad, USA. The cycling program was as follows: 10 min at 94°C; 10 cycles at 95°C for 20 s and 61–55°C for 60 s; 27 cycles at 95°C for 20 s and 55°C for 60 s; and 30 s at 25°C (Wang et al., 2021a). Individual samples were amplified in a 5 μL reaction system in a 96-well plate, and fluorescence detection of the reaction was performed using an Omega Fluorostar scanner; the data were analyzed using KlusterCaller 3.4.1 software, and the assay data were visualized using SNPviewer 2.0 software (Zhang et al., 2020, 2021).

### 2.4 KASP marker screening, genetic diversity analysis, and fingerprint construction

GenAlEx (version: 6.51b2) software was used to calculate the number of observed alleles (No), number of effective alleles (Ne), observed heterozygosity (Ho), expected heterozygosity (He), and Shannon’s diversity index (I) (Peakall and Smouse, 2012). PowerMarker 3.25 software was used to calculate the polymorphism information content (PIC) and minor allele frequency (MAF) (Liu and Muse, 2005). Neighbor-joining (NJ) trees were constructed based on candidate core and core SNP markers using MEGA 7.0 software. SNP-typing data were imported into STRUCTURE (version 2.3.1) software for analysis, with the K set from 1 to 20, MCMC set to 100,000, and length of the burn-in period set to 500,000; analyses were performed 10 times for each K-value (Falush et al., 2003). Structure Harvester was used to determine the optimal K-value (Evanno et al., 2005). The Q-value matrix was exported using CLUMPP 2.0 software and visualized using the R package poppelper (Jakobsson and Rosenberg, 2007). GenALEX (version: 6.51b2) software was used to calculate the genetic distance matrix and generate a PCA plot (Peakall and Smouse, 2012). Fingerprints were constructed based on candidate core SNP markers and core SNP markers using Perl scripts (Yang et al., 2022).

## 3 Results

### 3.1 Whole-genome resequencing data

In this study, 60 strains of *G. frondosa* were re-sequenced, and approximately 1.4 Gb of clean data per sample were obtained (Table 2 and Supplementary Table 1). After removing the low-quality sequences, a total of 575,355,502 high-quality reads were generated, and the average GC content of the Clean Data was 45.78%; the mean Q20 value was 96.53%, and the mean Q30 value was 91.29%. The size of the genome was 39.28 Mb; the mean genome coverage was 91.7%, the mean depth was 36 ×, and the average mapping rate of reads was 89.24%. The start and stop positions of the paired-end sequences on the reference genome were revealed, and the size and length distribution of the insert fragments after the fragmentation of the sample DNA was normal. The peak length of the insert fragments generally ranged from 300 to 400 bp, indicating that there were no abnormalities in the constructed libraries.

### 3.2 SNP type and distribution

The genomic variants of all the samples were filtered using GATK, and a collection of high-quality SNP variants of *G. frondosa* containing 2,125,382 SNP loci was obtained. The number of SNPs identified in each sample averaged 761,106, and the highest number of SNPs were identified in the Gf-4 strain (904,064 SNPs); the lowest number of SNPs were identified in the Gf-45 strain (730,920 SNPs). Further statistical analysis revealed that the number of SNPs per sample for Transitions was 599,093, the number of SNPs per sample for Transversions was 162,013, and the average Ts/Tv was 3.70. The number of SNPs for the heterozygosity type was 376,290, and the number of SNPs for the homozygosity type was 218,424. The C/T and G/A mutation types were the most common. Functional annotation results showed that these SNP mutations mainly occurred in the upstream region and coding sequence (CDS) region of genes, indicating that most of the high-quality SNP variants in *G. frondosa* were concentrated in the upstream regulatory sequences and CDSs. SNPs within the CDS region could be further categorized into synonymous mutations, non-synonymous mutations, termination codon gain, and termination codon deletion; the number of synonymous and non-synonymous coding mutations accounted for 62.19% and 36.60% of all mutations, respectively (Supplementary Table 2).

### 3.3 Population genetic analysis based on high-quality SNPs

**FIGURE 1:**
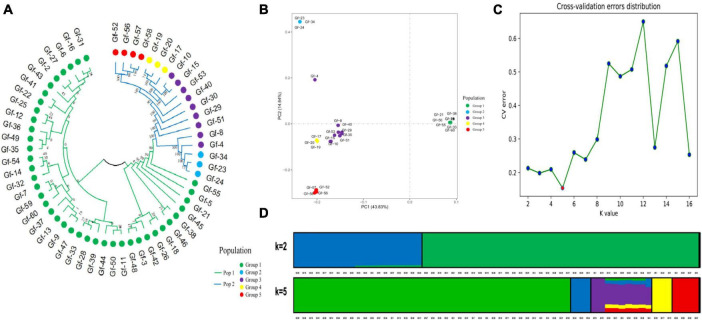
Population genetic analysis of 829,488 SNP loci from 60 *G. frondosa.* **(A)** Neighbor-joining phylogenetic tree using 829,488 SNPs in 60 *G. frondosa*. **(B)** Principal component analysis of the 60 *G. frondosa* with 829,488 SNPs. **(C)** Cross-validation errors distribution. **(D)** Population structure of the 60 *G. frondosa* using 829,488 SNPs based on the Admixture output for *K* = 5.

Phylogenetic trees provide insights into the evolutionary relationships, genetic distances, and evolutionary history of species. To clarify genetic relationships and the degree of differentiation among *G. frondosa* strains, an NJ analysis of 60 *G. frondosa* germplasm resources was conducted based on the obtained 829,488 high-quality SNP loci. These strains could be divided into two populations: Pop1 (the green branch) and Pop2 (the blue branch). Pop1 comprised the most strains (41) (Figure 1A). Population structure analysis of *G. frondosa* germplasm resources was performed using filtered high-quality SNPs (Supplementary Figure 1). The CV error was lowest when K = 5 (Figure 1C), and the lowest cross-validation value was 0.15424. The 60 *G. frondosa* germplasm resources could be further divided into five subgroups (Figure 1D). The three wild strains Gf-29, Gf-30, and Gf-40 from Shandong Province were nested within Group 3, and this was consistent with their observed geographical distributions.

The first principal component and the second principal component explained 43.83% and 14.64% of the variation in the data, respectively (Figure 1B). and the 60 germplasm resources of *G. frondosa* could be divided into two categories according to the first principal component; the second population could be further divided into four subgroups according to the second principal component, and Group 3 and Group 4 were closely clustered, which was consistent with the results of the cluster analysis and population structure analysis. Different degrees of variation were observed in the eight aerospace mutagenic strains and the original strain Gf-31, but they were nested within subgroup Group 1; the three cultivated strains Gf-31, Gf-9, and Gf-37 introduced from Japan were nested within subgroup Group 1, indicating that strains from the same country were closely related and that the results of our cluster analysis were reliable.

### 3.4 Development of core collection

**FIGURE 2:**
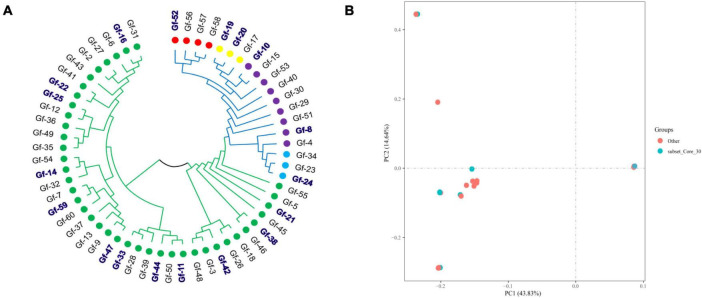
The developed core collection based on 829,488 high-quality SNPs using Core Hunter. **(A)** Distribution of the core collection in the cluster analysis results; the blue and bold area represents the core germplasm resource population. **(B)** The core collection population was evaluated using PCA.

To provide a subset of representative germplasm collections for the selection of parents in *G. frondosa* breeding, we developed a core collection of *G. frondosa* germplasm collections using the 829,488 high-quality SNPs. The core collection was selected using Core Hunter software with the parameters set at 19 different values (5%, 10%, 15%, 20%, 25%, 30%, 35%, 40%, 45%, 50%, 55%, 60%, 65%, 70%, 75%, 80%, 85%, 90%, and 95%). Genetic diversity indicators for the core collection are important for assessing the level of genetic diversity of the selected core collection. The core collection included 18 representative *G. frondosa* germplasm resources, which captured 30% of the total number of original germplasm collections with 100% allele coverage. The observed number of alleles (Na), effective number of alleles (Ne), Shannon’s information index (I), observed heterozygosity (Ho), expected heterozygosity (He), polymorphic information content (PIC), and Nei’s genetic diversity (H) were 1.9995, 1.5773, 0.5239, 0.3814, 0.3471, 0.2805, and 0.3471, respectively (Supplementary Table 3). The results of principal component analysis and cluster analysis showed that the core collection constructed in this study was basically consistent with the distribution of the original germplasm (Wang et al., 2021b), indicating that the core collection constructed under a 30% sampling ratio was representative of the genetic variation in the original collection. Principal component analysis was used to assess the core collection population, the results revealed that representative germplasms are present within all five subgroups, with green representing the core collection population and red representing the non-core collection population (Figure 2). This study will aid future innovation, as well as the utilization of *G. frondosa* germplasm resources, including the efficient selection and breeding of *G. frondosa*.

### 3.5 KASP marker design and screening

A set of standard processes based on the SNP variant database, including SNP filtering, KASP marker design, fingerprint construction, and population genetic analysis, were implemented to develop KASP markers. First, SNP sites conserved in sequences greater than 50 bp before and after the DNA strand on the chromosome were extracted and filtered to obtain 16,594 SNP sites; GATK software was used to obtain high-quality SNPs with various criteria (average coverage depth > 5 ×, MAF > 0.05, AverageQ > 30, minimum integrity > 0.9, and the SNP is a double-allele variant), which yielded 3,477 SNP sites. Sequences 100 bp upstream and downstream of the SNP markers were aligned to the reference genome using BLAST software (version 2.10.1+). Markers located at multiple positions in the alignment were removed, which yielded 2,916 SNP sites. Markers with a PIC > 0.20 were retained, and this yielded 1,706 high-quality SNP locus variants (Supplementary Table 4).

**FIGURE 3:**
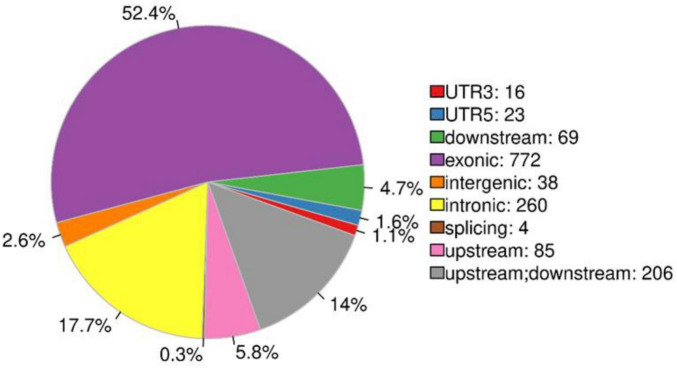
Distribution of SNP markers; different colors indicate the percentage of regions containing markers.

The primers for amplifying the SNP markers were designed using Primer 3 software and then transformed into KASP markers; a total of 1,473 markers were successfully designed, with a transformation rate of 86.34% (Supplementary Table 5). A total of 772 SNP markers were identified in the exon region, which accounted for 52.4% of all KASP markers (Supplementary Table 6), 260 SNP markers were distributed in the intron region, which accounted for 17.7% of all KASP markers; and 206 SNP loci were distributed in the gene regulatory region 2 kb upstream and downstream of the gene, which accounted for 14% of all KASP markers (Figure 3).

A combination of physical location, PIC, MAF, observed heterozygosity, and Missing values of *G. frondosa* genotypes were used to identify candidate core SNPs. Based on the KASP analysis of 60 *G. frondosa* germplasm resources, KASP genotyping experiments were carried out on 772 SNP markers distributed in the exon region, and 50 high-quality candidate core SNPs were obtained; these candidate core SNP markers can be used to distinguish among *G. frondosa* populations (Supplementary Table 7).

**FIGURE 4:**
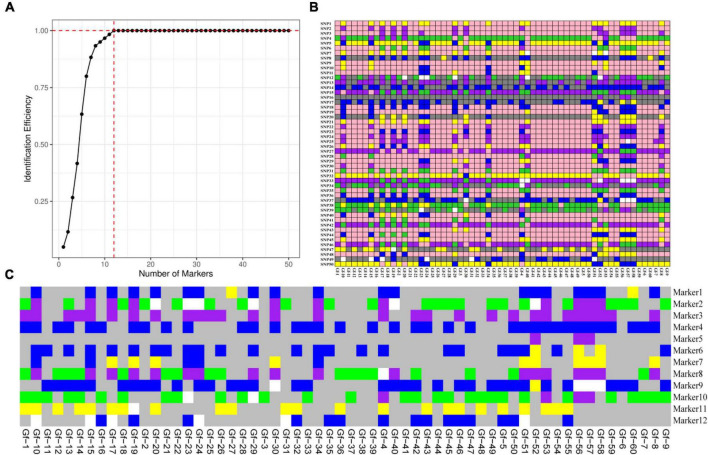
Analysis of genetic diversity indexes based on a core SNP marker set of 60 *G. frondosa* germplasm resources. **(A)** Polymorphic information content (PIC). **(B)** Minor allele frequency (MAF). **(C)** Observed heterozygosity. **(D)** Occurrence of missing values.

According to the saturation curve analysis of marker identification efficiency, 12 core markers with high detection rates and high polymorphisms that could distinguish all varieties in this experiment were selected. The PIC values of the 12 core SNP markers were in the range of 0.269 to 0.375, with an average value of 0.337, and 10 out of the 12 core markers had PIC values greater than 0.3. The average MAF value of the 12 markers was 0.343 with a range of 0.2–0.492. The average observed heterozygosity was 0.563, and 83% of the core SNP markers had missing values < 0.10, indicating that the 12 core SNP markers were sufficiently polymorphic (Figure 4). Detailed information on the 12 core SNP markers (marker name, location, variant type, and primer sequences) is provided in Table 3.

**TABLE 3:** KASP primer names, positions, variant types, and sequences for the 12 core SNP markers.

ID	Chr_Position	Primer sequences (5′–3′)	Variant type
Marker1	LUGG01000002.1_2880791	F1:GAAGGTGACCAAGTTCATGCTACCACAACAGCGCAGCAGTA	A/G
		F2:GAAGGTCGGAGTCAACGGATTCCACAACAGCGCAGCAGTG	
		R:CAACCGCTCGTCTAATAATTCTCC	
Marker2	LUGG01000003.1_1987918	F1:GAAGGTGACCAAGTTCATGCTCGACTGCGTCTTGTATTTCTCTT	A/G
		F2:GAAGGTCGGAGTCAACGGATTCGACTGCGTCTTGTATTTCTCTC	
		R:GAGATGACGAGGCAGTGAATGAC	
Marker3	LUGG01000004.1_346179	F1:GAAGGTGACCAAGTTCATGCTGTATTTCGTGCAGTGCTCCTTA	T/C
		F2:GAAGGTCGGAGTCAACGGATTTTTCGTGCAGTGCTCCTTG	
		R:AGGAGGAGATGAAGGAAGTTTGTT	
Marker4	LUGG01000006.1_718221	F1:GAAGGTGACCAAGTTCATGCTCCTTCCTCCAGCACAAGCC	G/A
		F2:GAAGGTCGGAGTCAACGGATTTCCTTCCTCCAGCACAAGCT	
		R:TGACTATCTGCGGGATACTGAAGTT	
Marker5	LUGG01000013.1_1509986	F1:GAAGGTGACCAAGTTCATGCTGTCTCGTCATCCGAGTTGTCG	G/A
		F2:GAAGGTCGGAGTCAACGGATTAGTCTCGTCATCCGAGTTGTCA	
		R:AGAGTGATGTGATGTTGTCTGAGCA	
Marker6	LUGG01000017.1_156103	F1:GAAGGTGACCAAGTTCATGCTGTGCTTGCTTACCACAGCTTCTAT	T/C
		F2:GAAGGTCGGAGTCAACGGATTGTGCTTGCTTACCACAGCTTCTAC	
		R:GTTGCTCACTATCAGACAGTTTAAGGG	
Marker7	LUGG01000022.1_499394	F1:GAAGGTGACCAAGTTCATGCTTCCCTTCCTTCTAGCCTTGACC	C/A
		F2:GAAGGTCGGAGTCAACGGATTTCCCTTCCTTCTAGCCTTGACA	
		R:GCCTCGAGATCTGGAATTTTGTAG	
Marker8	LUGG01000003.1_1053539	F1:GAAGGTGACCAAGTTCATGCTTGACCAGCTACTCTTGCAGGG	C/T
		F2:GAAGGTCGGAGTCAACGGATTCTGACCAGCTACTCTTGCAGGA	
		R:TACAGCTGGATGGGAGTCGTCA	
Marker9	LUGG01000003.1_2042709	F1:GAAGGTGACCAAGTTCATGCTAGGTTTACAGGAGCTGTTGTAGATATG	C/T
		F2:GAAGGTCGGAGTCAACGGATTCAGGTTTACAGGAGCTGTTGTAGATATA	
		R:TCTAAGGTTTTCAACAAAGTCAGCG	
Marker10	LUGG01000002.1_1446577	F1:GAAGGTGACCAAGTTCATGCTAGGCGACGGTGTCATGAGG	C/T
		F2:GAAGGTCGGAGTCAACGGATTGAGGCGACGGTGTCATGAGA	
		R:CTGCTGAACCTCTGGAGACTCGTAT	
Marker11	LUGG01000023.1_401039	F1:GAAGGTGACCAAGTTCATGCTCCAACTCTTGATCTACCTGCCAAT	A/G
		F2:GAAGGTCGGAGTCAACGGATTCCAACTCTTGATCTACCTGCCAAC	
		R:TAGAGTCGCGCAAATTGGAGGT	
Marker12	LUGG01000033.1_182680	F1:GAAGGTGACCAAGTTCATGCTGCGCAAAATATGCTTGCCTC	C/A
		F2:GAAGGTCGGAGTCAACGGATTAGCGCAAAATATGCTTGCCTA	
		R:CGGCATTTGAAAGCTCGAGAA	

### 3.6 Genetic diversity analysis based on KASP markers and fingerprint construction

**FIGURE 5:**
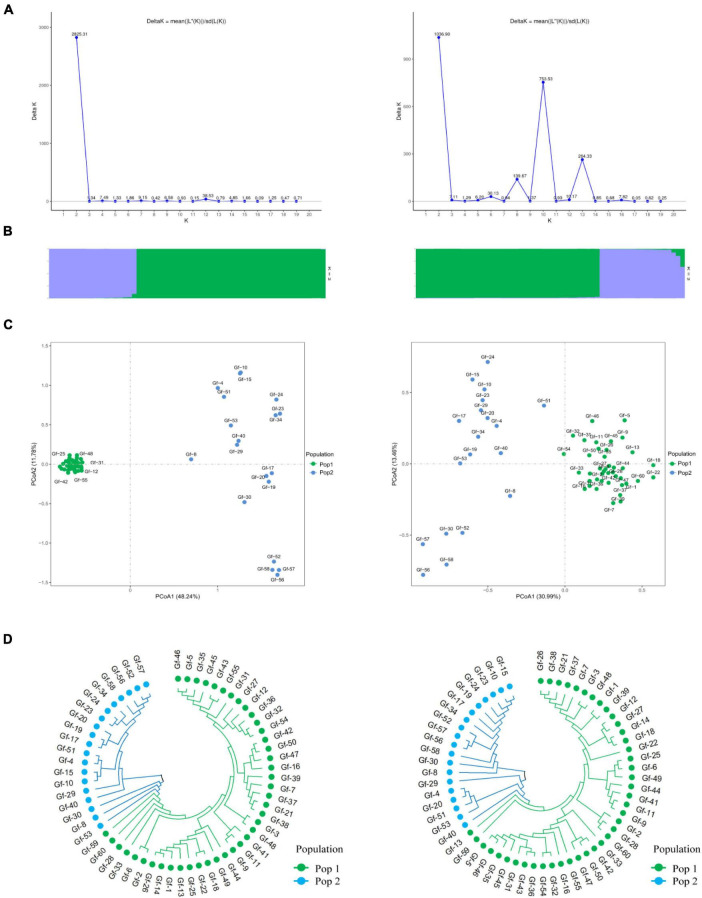
Population diversity analysis of 60 *G. frondosa* germplasm resources using 50 candidate core SNPs (left) and 12 core SNPs (right). **(A)** Delta K values. **(B)** Population structure of the germplasm resources inferred at *K* = 2. **(C)** Principal component analysis (PCA) of 60 *G. frondosa* germplasm resources. **(D)** Neighbor-joining tree showing a dendrogram of 60 *G. frondosa* germplasm resources. Green and blue color indicated two different populations.

**FIGURE 6:**
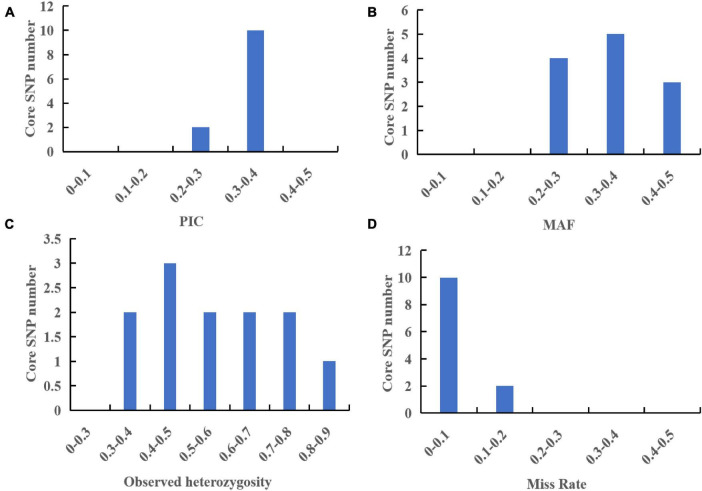
**(A)** Saturation curve of marker identification efficiency. The horizontal axis indicates the number of markers, and the vertical axis indicates the identification efficiency. **(B)** Fingerprint construction based on 50 candidate core SNP makers. **(C)** Fingerprint construction based on 12 core SNP makers; the horizontal axis shows samples, and the vertical axis shows molecular markers; pure genotypes C/C, A/A, T/T, G/G are shown in yellow, green, blue, and purple respectively; the heterozygous genotype in the core marker is gray; the pink represents the heterozygous genotype in the non-core marker; and the deletion genotype is white.

To further evaluate the discriminative ability of candidate core and core SNP markers in *G. frondosa* germplasm resources, 50 candidate core SNP markers and 12 core SNP markers were used for population genetic diversity analysis (Figure 5 and Supplementary Figures 2, 3). Assessment of the relationships among the 60 *G. frondosa* germplasm resources using STRUCTURE showed that the optimal K-value was 2, which divided the 60 *G. frondosa* germplasm resources into two populations using either the 50 candidate core SNPs or the core SNP marker set. PCA and the NJ trees based on the genotyping data were consistent with the results of the population genetic structure analysis when the two SNP marker sets were used. The 50 candidate core markers and 12 core markers, based on the first principal component, can both divide the 60 Grifola frondosa strains into two major groups: Pop1 (41) and Pop2 (19) (Figure 5C). The results thus indicate that the core SNP marker set was highly representative, and its discriminatory power was similar to that of the 50 candidate core SNPs. Based on the 50 candidate core SNPs and the 12 newly developed core SNP markers, we constructed fingerprints of 60 *G. frondosa* germplasm resources (Figure 6), our findings highlight the efficiency and accuracy of genotyping with the core SNP markers. SNP fingerprinting provided an accurate, rapid, convenient, and efficient method for the identification of *G. frondosa* germplasm resources.

## 4 Discussion

Germplasm resources are essential for the breeding of edible mushrooms, and the quality of the varieties produced by breeding largely depends on studies of the genetic diversity of these germplasm resources. Techniques for evaluating the genetic diversity of edible mushrooms germplasm resources mainly include morphological markers, cytological markers, biochemical markers, and molecular markers (Wang et al., 2019; Mahmoodi et al., 2021). Molecular markers are nucleotide sequence variants that provide direct indicators of genetic polymorphism at the DNA level. A range of DNA molecular markers, including ITS, RFLP, RAPD, AFLP, ISSR, SRAP, and SCAR markers, have been used for the characterization of *G. frondosa* germplasm resources (Zhang et al., 2009; Wen et al., 2011; Yang et al., 2016; Wang et al., 2019). No studies have resequenced *G. frondosa* germplasm resources nor developed SNP markers based on resequenced data.

In this study, 60 *G. frondosa* germplasm resources were re-sequenced, and a total of 84 Gb of re-sequencing data were obtained, with an average sequencing depth of 36 ×. After mutation detection and filtering, 829,488 high-quality SNP loci were obtained for subsequent analysis. Phylogenetic analysis, PCA, and population structure analysis all showed that the 60 germplasms could be divided into two populations and five subgroups, which is consistent with the results of previous studies. Yang et al. used RAPD and ISSR molecular markers to analyze the genetic diversity of domestically cultivated *G. frondosa* strains and divided 40 *G. frondosa* strains into five subgroups (Yang et al., 2016). Wang et al. analyzed the genetic diversity of 42 *G. frondosa* strains by ISSR and SRAP molecular markers, and divided them into 6 groups, which provided a basis for parent selection in hybrid breeding at molecular level (Wang et al., 2019). Phylogenetic analysis revealed that three of the wild strains, Gf-29, Gf-30, and Gf-40, were nested within Group 3, which has low genetic diversity. This might stem from the proximity of the collection sites of the wild strains. The three cultivated strains Gf-31, Gf-9, and Gf-37 introduced from Japan were nested within Group 1, suggesting that strains from the same country were more closely related. The whole-genome re-sequencing data provided new and more comprehensive evidence for the classification of *G. frondosa*, clarified the genetic relationships among *G. frondosa* in China, and provided substantial and valuable genomic resources, which has implications for the improvement of *G. frondosa* germplasm and molecular breeding. Based on the 829,488 high-quality SNP loci obtained by genome-wide resequencing, 18 representative core germplasms were screened by Core Hunter software. According to the genetic diversity index, PCA, and cluster analysis, the core collection comprised a representative sample of the original germplasm population.

We used a high-throughput SNP genotyping platform to develop a core set of 50 candidate SNP markers from the resequencing data of the 60 *G. frondosa* germplasm resources. DNA fingerprinting is a simple and economically efficient method that aims to identify large numbers of varieties with few markers. A total of 12 core markers with high detection rates and high levels of polymorphism that could distinguish all varieties in this experiment were selected. Phylogenetic analysis, PCA, and population structure analysis based on candidate core and core SNP markers showed that 60 *G. frondosa* germplasms could be divided into two populations, and the clustering results were highly consistent with the population re-sequencing data.

Study of the *G. frondosa* germplasm resources revealed that the *G. frondosa* population has high genetic variation and a high diversity index; this core collection of *G. frondosa* thus has major implications both for the economic value of *G. frondosa* and for future research. Additional analyses are needed to maximize the economic and scientific value of this core collection. First, the materials examined in this study were mainly derived from cultivated species in the main *G. frondosa*-producing areas in China. The genetic background of *G. frondosa* germplasm is relatively narrow; wild germplasm contains a large number of excellent alleles, yet these have not been effectively applied. More research on wild strains is needed. There is also a need to finely characterize these strains, as well as conduct analyses of phenotypic traits and biological properties. In the future, the genes related to important agronomic traits of *G. frondosa* can be further screened by combining GWAS and phenotypic data, and identification of the genes that regulate genetic variation in phenotypic traits is needed to facilitate the fine-mapping of candidate functional regions. Second, *G. frondosa* is a rare edible and medicinal mushroom with a complex genetic background, and the chromosomal genome assembly has not yet been generated. A high-quality reference genome is essential for studies of population variation and genome evolution. There are a large number of mutations in the genome, including SNPs, InDels, CNVs, and SVs, and these variatns can facilitate the development of specific molecular markers. The materials selected for this study are mostly cultivated species from the main production areas of *G. frondosa* in China, with a relatively limited genetic background. The number of wild germplasm resources containing rich excellent alleles is limited. In the future, further line breeding should pay more attention to the collection, development, and research of wild strains.

In sum, we constructed a core collection based on whole-genome resequencing and developed a set of core SNP markers that can be used to distinguish among the main cultivated, wild, and mutated varieties of *G. frondosa* in China. Genetic diversity and population structure analyses were conducted based on the candidate core and core SNP markers, and the results showed that the screened candidate core and core SNP markers could be used to distinguish among all the experimental materials in the phylogenetic tree. DNA fingerprints were constructed based on the screened SNP markers, and this provides an accurate, rapid, convenient, and efficient method for the identification of germplasm resources. These results are important for the preservation and utilization of *G. frondosa* germplasm resources, variety identification, and the protection of new varieties.

## Data availability statement

All the genomic sequence data sets are available in the NCBI Sequence Read Archive under the accession number PRJNA1034769.

## Author contributions

BD: Data curation, Project administration, Writing−original draft, Writing−review and editing. ZX: Supervision, Validation, Writing−review and editing. ML: Supervision, Validation, Writing−review and editing. GZ: Supervision, Writing−review and editing. GW: Supervision, Writing−review and editing. YZ: Supervision, Writing−review and editing. ZT: Conceptualization, Funding acquisition, Project administration, Resources, Supervision, Writing−review and editing.

## Footnotes

1
https://ftp.ncbi.nlm.nih.gov/genomes/genbank/fungi/Grifola_frondosa/latest_assembly_versions/GCA_001683735.1_ASM168373v1/

## Supplementary material

Supplementary Figure 1 Population structure of the 60 *G. frondosa* using 829,488 SNPs based on the Admixture output from *K* = 2 to *K* = 10.

Supplementary Figure 2 Population genetic structure analysis of 60 germplasm resources of *G. frondosa* using 50 candidate core SNPs output from K2 to K10.

Supplementary Figure 3 Population genetic structure analysis of 60 germplasm resources of *G. frondosa* using 12 core SNPs output from K2 to K10.
