# Supplementary figures and images for: Establishment and application of a SNP molecular identification system in Grifola frondosa
Source: Front Microbiol. 2024 Aug 8;15:1417014. doi: 10.3389/fmicb.2024.1417014 (PMC11340509; doi:10.3389/fmicb.2024.1417014)

# Population Structure

k=2

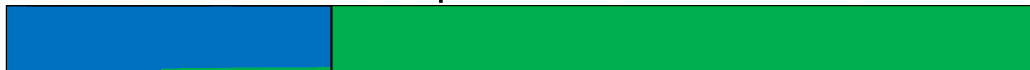

k=3

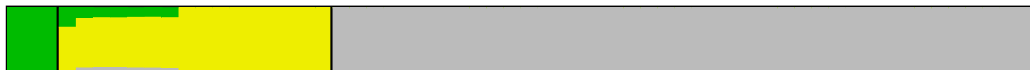

k=4

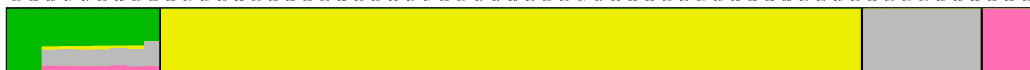

k=5

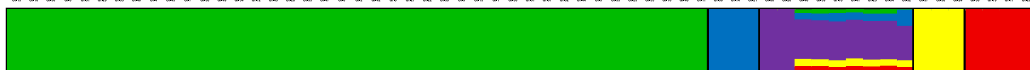

k=6

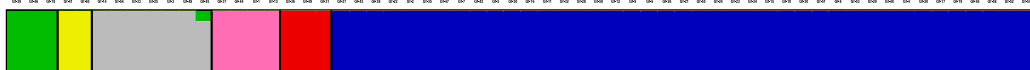

k=7

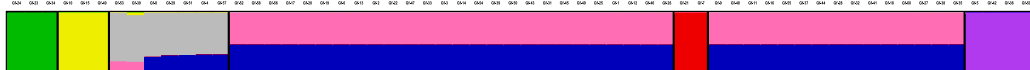

k=8

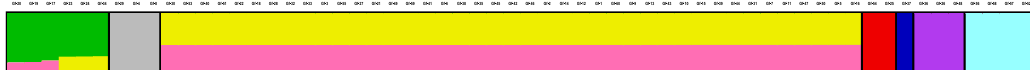

k=9

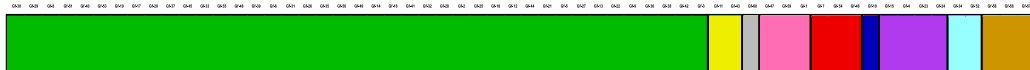

k=10

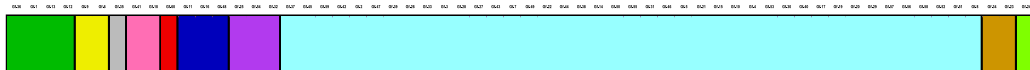

Supplement: Supplementary Figure 1 — Population structure of the 60 G. frondosa using 829,488 SNPs based on the Admixture output from K = 2 to K = 10. [file Data_Sheet_1.PDF]

K = 2

K = 3

K = 4

K = 5

K = 6

K = 7

K = 8

K = 9

K = 10

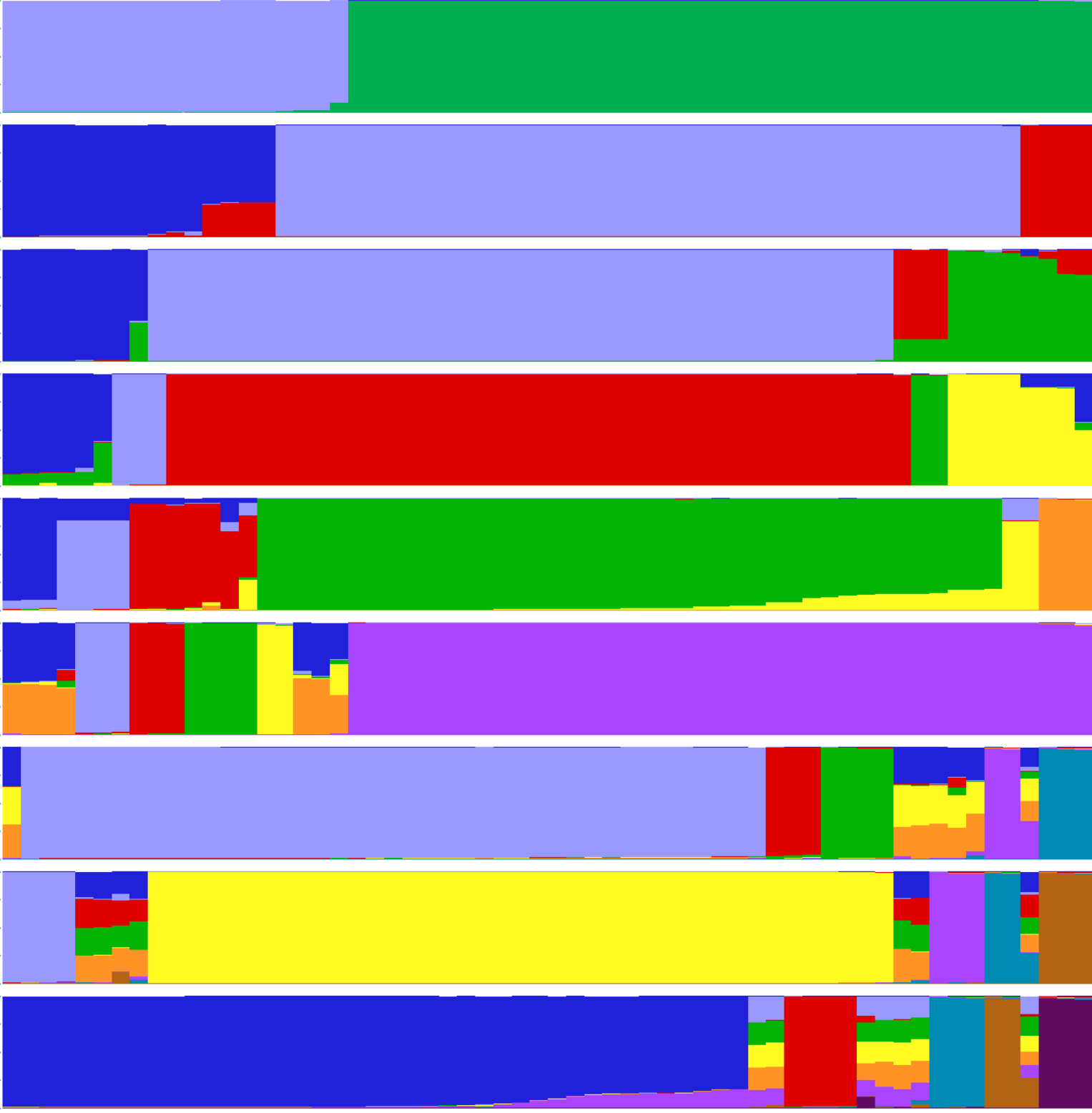

Supplement: Supplementary Figure 2 — Population genetic structure analysis of 60 germplasm resources of G. frondosa using 50 candidate core SNPs output from K2 to K10. [file Data_Sheet_2.PDF]

K = 2

K = 3

K = 4

K = 5

K = 6

K = 7

K = 8

K = 9

K = 10

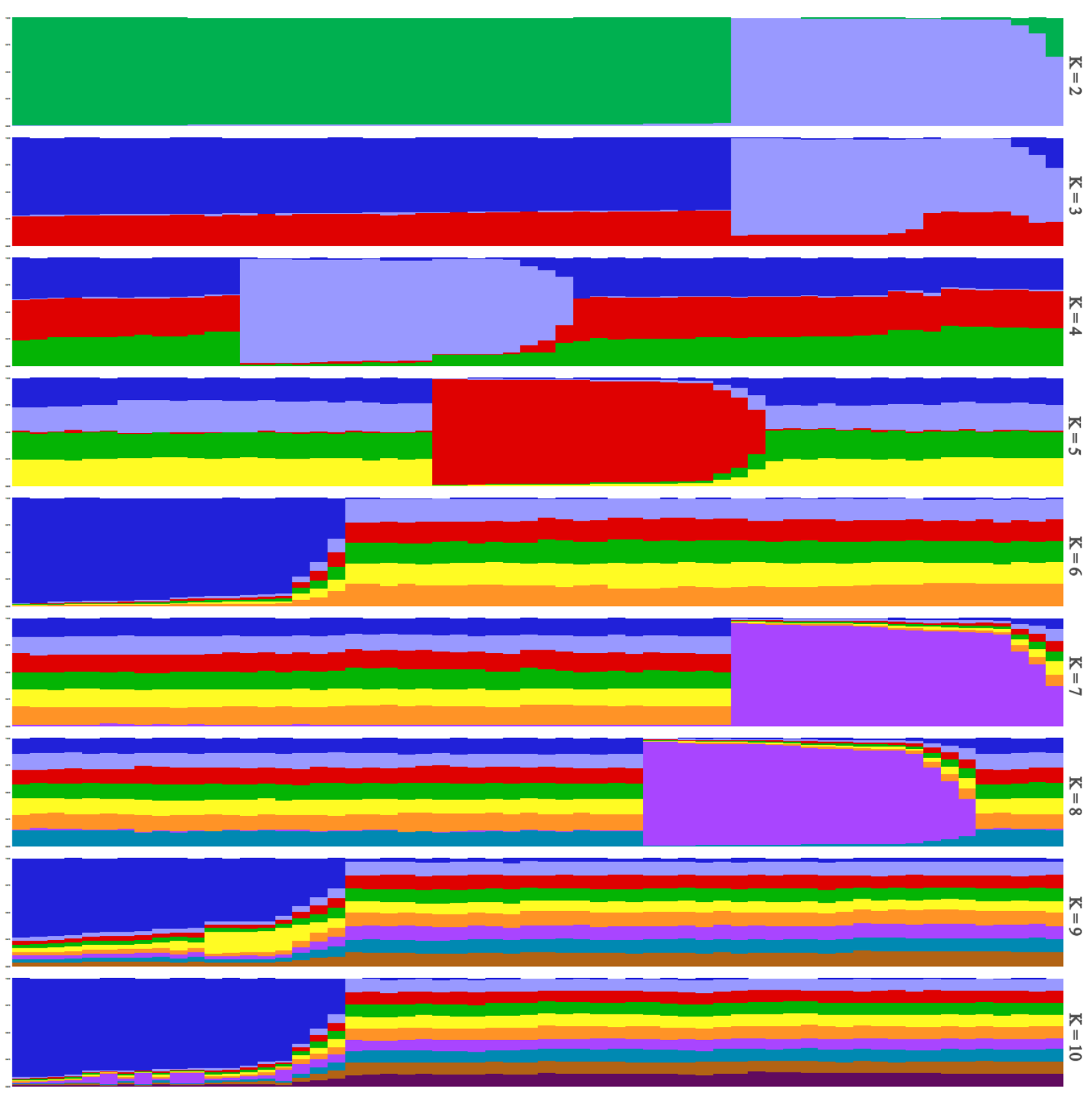

Supplement: Supplementary Figure 3 — Population genetic structure analysis of 60 germplasm resources of G. frondosa using 12 core SNPs output from K2 to K10. [file Data_Sheet_3.PDF]
